# Glycolysis-Derived Compounds From Astrocytes That Modulate Synaptic Communication

**DOI:** 10.3389/fnins.2018.01035

**Published:** 2019-01-23

**Authors:** Carlos-Alberto Gonçalves, Letícia Rodrigues, Larissa D. Bobermin, Caroline Zanotto, Adriana Vizuete, André Quincozes-Santos, Diogo O. Souza, Marina C. Leite

**Affiliations:** Department of Biochemistry, Federal University of Rio Grande do Sul (UFRGS), Porto Alegre, Brazil

**Keywords:** astrocyte, glycolysis, GSH, lactate, methylglyoxal, neurotransmission

## Abstract

Based on the concept of the tripartite synapse, we have reviewed the role of glucose-derived compounds in glycolytic pathways in astroglial cells. Glucose provides energy and substrate replenishment for brain activity, such as glutamate and lipid synthesis. In addition, glucose metabolism in the astroglial cytoplasm results in products such as lactate, methylglyoxal, and glutathione, which modulate receptors and channels in neurons. Glucose has four potential destinations in neural cells, and it is possible to propose a crossroads in “X” that can be used to describe these four destinations. Glucose-6P can be used either for glycogen synthesis or the pentose phosphate pathway on the left and right arms of the X, respectively. Fructose-6P continues through the glycolysis pathway until pyruvate is formed but can also act as the initial compound in the hexosamine pathway, representing the left and right legs of the X, respectively. We describe each glucose destination and its regulation, indicating the products of these pathways and how they can affect synaptic communication. Extracellular L-lactate, either generated from glucose or from glycogen, binds to HCAR1, a specific receptor that is abundantly localized in perivascular and post-synaptic membranes and regulates synaptic plasticity. Methylglyoxal, a product of a deviation of glycolysis, and its derivative D-lactate are also released by astrocytes and bind to GABA_A_ receptors and HCAR1, respectively. Glutathione, in addition to its antioxidant role, also binds to ionotropic glutamate receptors in the synaptic cleft. Finally, we examined the hexosamine pathway and evaluated the effect of GlcNAc-modification on key proteins that regulate the other glucose destinations.

## Aim

Glucose is the major energetic source of neural cells. In addition to providing ATP via the glycolytic pathway, glucose provides metabolites that are key to brain activity, such as glutamate and NADPH for lipid and glutathione (GSH) synthesis, as well as recycling of ascorbic acid. This short review will focus on glucose-derived compounds from astrocytes that modulate neurotransmission, in addition to providing energetic and substrate replenishment for brain activity. Glutamate, for example, is the main excitatory neurotransmitter and originates from astroglial glucose, as it is synthetized *de novo* from alpha-ketoglutarate. Two specific astrocyte enzymes, pyruvate carboxylase and glutamine synthetase, are necessary for this process. We will restrict this review to the role of glucose-derived compounds (arising directly from the glycolytic pathway in the cytoplasm of astroglial cells) that modulate synaptic receptors or transporters by binding to them, such as lactate, methylglyoxal, and GSH. Moreover, we will review the regulatory role of uridine diphosphate-*N*-Acetylglucosamine (UDP-GlcNAc), which covalently regulates several astrocyte proteins, including glucose metabolism enzymes and related transcription factors, which in turn modulate synaptic communication.

## Introduction

**FIGURE 1:**
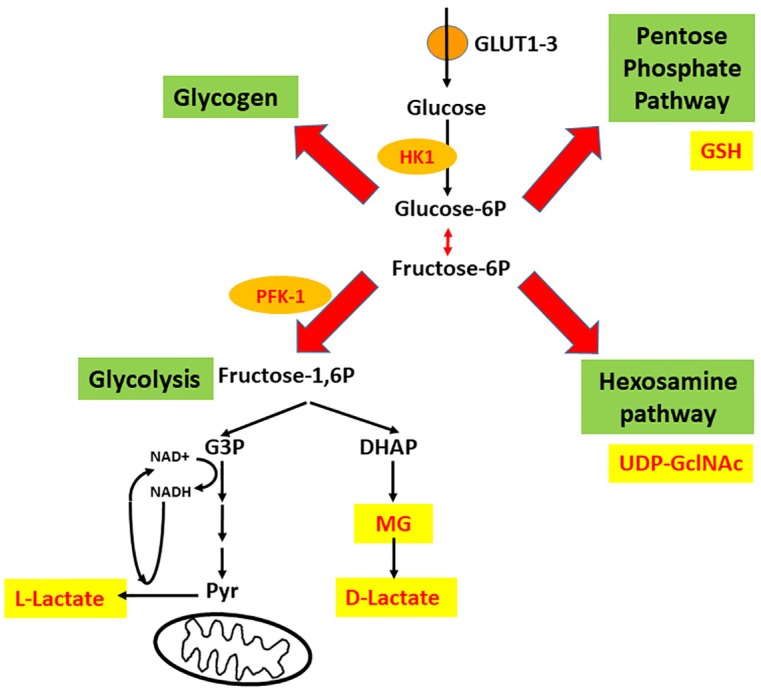
Four intracellular destinations of glucose that suggest an intersection in “X.” Glucose enters astrocytes mainly via GLUT1, and neurons mainly via GLUT3 and is trapped by phosphorylation (catalyzed by hexokinase 1, HK1). Afterward, four destinations are possible; these form a crossroads in the shape of an X, where glycogen synthesis and the pentose phosphate pathway (PPP) are the left and right arms of the X, and glycolysis (until pyruvate) and the hexosamine pathway (HP) are the left and right legs of the X. The deviation of glycolysis that generates methylglyoxal (MG) is also indicated. PFK-1, phosphofructokinase-1; G-3P, glyceraldehyde 3-phosphate; DHAP, dihydroxyacetone phosphate.

Preliminarily, it is important to highlight the importance of astrocytes in the synapse, particularly in glucose metabolism. Although there is no doubt about the significance of blood glucose for brain activity, the mode of entry of glucose to the brain and its cell distribution are still debated (e.g., Lundgaard et al., 2015; Barros et al., 2017). Once inside the cell, glucose is phosphorylated on carbon 6 by hexokinase (HK), generating glucose-6P, which is converted to fructose-6P via the action of an isomerase. These two compounds (glucose-6P and fructose-6P) are used in at least two different pathways. It is possible to propose a metaphorical “X” intersection of these reactions, as illustrated in Figure 1, to describe the destinations of glucose in the neural cells. Glucose-6P can be converted to fructose-6P, but can also be used for glycogen synthesis or in the pentose phosphate pathway (PPP) (left and right arm of the X, respectively). On the other hand, fructose-6P continues through the glycolysis pathway until pyruvate is formed, but can also act as the initial compound in the hexosamine pathway, as represented by the left and right legs of the X, respectively.

### Glucose Transport and Phosphorylation

Before discussing the destinations of glucose, it is important to understand how it enters the neural cells, via the glucose transporter (GLUT) and sodium-glucose co-transporter (SGLT), which are blocked by phloretin and phlorizin, respectively (Shah et al., 2012). GLUTs are passive and bidirectional transporters. GLUT1 is the main isoform found in cells forming the blood-tissue barriers (endothelial and choroid plexus), astrocytes and the ependymal cells lining the cerebral ventricles. In addition to glucose, GLUT1 transports dehydroascorbic acid (the oxidized form of vitamin C) (Rivas et al., 2008) and glucosamine (Chopra, 2004). At the blood-brain barrier (BBB), GLUT1 works at about one-third of maximal capacity under basal conditions (Leybaert et al., 2007). Neurons express mainly GLUT3, although some neurons in the rodent brain also express the insulin-sensitive GLUT4 (in the hippocampus, the cerebellum and the hypothalamus) (Choeiri et al., 2002) and SGLT1 (in the hippocampus and the cerebral cortices) (Yu et al., 2010). However, glucose transportation by these carriers is not considered a rate-limiting step in brain energy metabolism. In contrast, the next step, glucose phosphorylation by hexokinase, represents the rate-limiting step.

Notably, more than 90% of non-fenestrated capillary brain vessels are covered by astrocytic end-feet (Jukkola and Gu, 2015). Moreover, the tight junctions between endothelial cells (responsible for non-fenestration) are actively regulated by astrocyte signals (Ballabh et al., 2004). These aspects indicate the importance of astrocytes in glucose distribution. However, this does not mean that glucose needs to pass through astrocytes to reach neurons. In fact, after crossing endothelial cells via GLUT 1, glucose can reach neurons directly via GLUT3, because there is room for molecular diffusion, since astrocytes form gap junctions among themselves instead of tight junctions.

Glucose phosphorylation on C_6_, catalyzed by HK, is the first rate-limiting step of glycolysis. All three isoforms of HK (of low Km) are present in brain tissue, but HK1 is the most abundant isoform in neurons and astrocytes and it is assumed to be “the brain hexokinase.” HKs 1 and 2 bind to the outer mitochondrial membrane by a hydrophobic sequence at their N-terminal, close to the pore that allows ATP output (Pastorino and Hoek, 2008). The activation of Akt kinase (or inhibition of glycogen synthase kinase 3, GSK-3) favors the binding of HK to the mitochondria by a mechanism that is still unclear. Glucose-6P induces a conformational change of the HK, displacing it from the mitochondria and decreasing its activity. Glucose-6P acts as a non-competitive inhibitor of HK, which under basal conditions is predominantly inhibited (DiNuzzo et al., 2015).

### PFK-1 Catalyzes the Other Rate-Limiting Step of Glycolysis

Glucose-6P is isomerized to fructose-6P, which in turn is converted to fructose-1,6-bis-phosphate (F1,6BP, see Figure 1). This reaction is the second rate-limiting step of glycolysis and is catalyzed by PFK-1. HK has been reported to be higher expressed in neurons than in astrocytes (Lundgaard et al., 2015); however, the activity and regulation of PFK-1 in astrocytes suggest a higher glycolytic activity in these cells (Bolaños et al., 2010). PFK-1 is allosterically downregulated by metabolites ATP, citrate and long-chain fatty acids (Jenkins et al., 2011) and upregulated by fructose 2,6 bisphosphate (F2,6BP) (Mor et al., 2011). Moreover, lactate, at least in muscle cells, is able to disarrange the tetrameric structure and reduce the enzymatic activity of PFK-1 (Costa Leite et al., 2007). F2,6BP, the main allosteric activator, is present in astrocytes at higher concentrations than in neurons. In fact, the enzyme responsible for the generation of F2,6BP from fructose-6P in brain tissue, 6-phosphofructo-2-kinase/fructose-2,6-bisphosphatase-3 (PFKFB-3), has lower expression levels in neurons due to elevated proteosomal degradation (Herrero-Mendez et al., 2009). PFKFB-3 is the target of several kinases, including Akt and AMP-activated protein kinase (AMPK; Marsin et al., 2000). AMPK is able to phosphorylate/activate PFKFB-3 as well as PFK-1 (Bartrons et al., 2018), indicating a direct regulatory role of AMP.

The lower activities of PFK-1 and PFKFB-3 in neurons suggest that glucose uptake could be conducted to the PPP to generate NADPH, which is required for the regeneration of GSH in these cells (Bolaños et al., 2010). Neurons have low concentrations of GSH (Dringen et al., 2005) and activity of γ-glutamyl cysteine ligase (GCL; Makar et al., 1994) when compared to astrocytes. Interestingly, oxidative stress-mediated S-glutathionylation of PFKFB-3 decreases its catalytic activity in cancer cells, redirecting the glycolytic flux to the PPP (Seo and Lee, 2014). Understanding the metabolic regulation of these three enzymes (HK1, PFK-1, and PFKFB-3) is important for comprehending the journey of glucose to pyruvate (including the passage through glycogen) or to ribulose-5 (PPP), as well as the effects of products of these pathways (lactate, methylglyoxal, and GSH) on synaptic communication. All these regulatory enzymes of glucose flow are direct or indirect targets of GlnNAcylation, which in turn depends on glucose flow itself, as we will discuss below.

## The Left Arm and Leg of Glucose Metabolism Modulate Synaptic Transmission Via Lactate

Lactate, directly derived from glucose or glycogen (in astrocytes), performs functions beyond energy supply. These functions are mediated by different mechanisms and newly presented pathways (already verified or still proposed), including a specific receptor and its signaling transduction pathways.

### The Lactate Receptor: For Every Orphan, a Family

The lactate receptor, initially known as GPR81, belongs to a family of G protein-coupled receptors (GPRs). It was first mapped via a genomic sequence database and then identified in the human pituitary gland (Lee et al., 2001). At that time, in the absence of a specific ligand, the receptor was considered an orphan. Later, the receptor was shown to be highly expressed in adipose tissue (Liu et al., 2009). The subsequent pharmacological characterization of L-lactate as a ligand for GPR81 was initiated, taking into account the similarity of GPR81 with other receptors from the GPR family, GPR109a and GPR109b, which also have β-hydroxybutyrate as a ligand. L-lactate inhibits lipolysis via GPR81 in adipocytes from human, mouse, and rat adipose tissue (Cai et al., 2008; Liu et al., 2009). The suggestion that lactate may act in a hormone-like manner comes from the demonstration of an insulin-dependent inhibition of lipolysis via GPR81 by Ahmed et al. (2010).

As all GPR ligands are hydroxy-carboxylic acids, the GPRs are now HCA receptors (HCARs; Ahmed et al., 2009; Blad et al., 2011; Offermanns, 2017). GPR89/HCAR1 has received more attention during recent years and has been implicated in inflammation and cancer signaling [for reviews, see (Haas et al., 2016; Offermanns, 2017)]. A compound present in fruits (Liu et al., 2012; Bergersen, 2015), 3,5-dihydroxybenzoic acid (DHBA), was identified as an agonist for HCAR1, inhibiting lipolysis in wild-type mouse adipocytes, but not in HCAR1 knocked-down adipocytes (Liu et al., 2012). The HCAR2 ligand 3-hydroxy-butyrate has been considered as an antagonist for the HCA1 receptor and has been used experimentally as such (Shen et al., 2015).

To characterize HCAR1 signaling pathways, a study using a cell line designed to express human HCAR1 (CHO-K1) showed activation of extracellular signal-regulated kinases (ERK1/2) via HCAR1 in response to lactate and DHBA and sensitivity to the G_i_ protein inhibitor pertussis toxin (Li et al., 2014). Moreover, the Gαγ subunit dissociated from the activated G_i_ protein was central in the regulation of HCAR1-activated ERK1/2 phosphorylation via extracellular Ca^2+^, protein kinase C (PKC), and insulin-like growth factor-1 receptor (IGF-1R) activation. Arrestin-2 and 3 had no effect on ERK1/2 activation, whereas HCAR1 internalization was dependent on arrestin-3 (Li et al., 2014). Supposed non-canonical actions of the HCAR1 receptor, i.e., without involving cyclic AMP (cAMP) reduction, have been proposed based on β-arrestin actions but still await future confirmation (Bergersen, 2015; Morland et al., 2015) and a different yet unknown receptor has also been suggested (Tang et al., 2014).

D-lactate, the stereoisomer of L-lactate, is produced at very low concentrations under physiological conditions from methylglyoxal (MG, see below). It is considered to be a partial agonist of the HCAR1 receptor with maximal stimulation significantly lower than that by L-lactate (Cai et al., 2008). As reported throughout this review, although some studies have shown D-lactate as a positive control for L-lactate HCAR1 signaling in neurons (Bozzo et al., 2013), many other studies have shown antagonistic (Tang et al., 2014), absent (Herrera-López and Galván, 2018), and controversial actions of the lactate enantiomer on neuroprotection and cognitive functions (Gibbs and Hertz, 2008; Castillo et al., 2015).

### Synaptic and Vascular Modulation by Lactate

**FIGURE 2:**
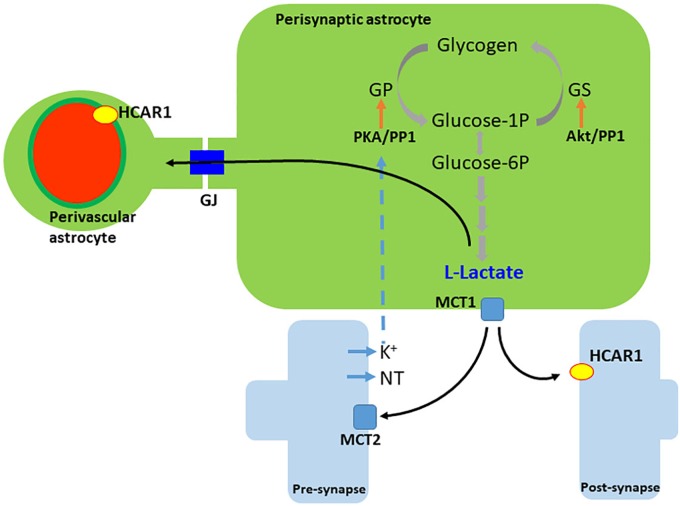
Generation and release of lactate in astrocytes. L-lactate is generated either from recently uptaken glucose or from glycogen. Neurotransmitters (NT), and/or extracellular K^+^, trigger glycogen breakdown until lactate, via cAMP/PKA signaling. Lactate leaves the cell by the monocarboxylate transporter 1 (MCT1) and enters neurons via the monocarboxylate transporter 2 (MCT2). Extracellular lactate also binds to hydroxy-carboxylic acid receptor 1 (HCAR1), which is found more abundantly in perivascular and post-synaptic membranes. Lactate travels among astrocytes through gap junctions (GJ). PKA, protein kinase A; GP, glycogen phosphorylase; GS, glycogen synthase; PP1, protein phosphatase 1.

The presence of the – at that time – orphan GPR81 was first demonstrated in brain tissue in 2001 (Lee et al., 2001). HCAR1 is located at synaptic membranes of excitatory synapses in the hippocampus and the cerebellum. It is located predominantly at postsynaptic sites, but it is also present in astroglial end-feet processes and endothelial membranes, indicating that energy metabolism is associated with synaptic function (Lauritzen et al., 2014). The vascular endothelial density of the receptor is twice the density at the astrocytic end-feet, suggesting a lactate control of cerebral blood flow (see Figure 2). The effect of physical exercise on the density of capillaries via HCAR1 in the sensorimotor cortex and more markedly in the hippocampus, was reproduced by daily subcutaneous injections of L-lactate (about 10 mM in the blood). The regulation of angiogenesis via HCAR1 and downstream Erk1/2 and Akt signaling resulting in vascular endothelial growth factor (VEGF) production were confirmed by the extensive expression of the receptor at perivascular pial and pericyte-like cells (Morland et al., 2017).

Even before the deorphanization of the GPR81, several non-metabolic actions of lactate have been reported in neural cells. Lactate increases the action potential frequency of glucose-sensing neurons at the ventromedial hypothalamic nucleus via K_ATP_ and chloride channels (Song and Routh, 2005). Another study using cortical astrocyte cultures and SH-SY5Y neuronal cells incubated with lactate showed increased brain-derived neurotrophic factor (BDNF) and inducible nitric oxide synthase (iNOS) expression in astrocytes but not in SH-SY5Y cells. However, the authors centered their discussion only on the energetic aspect of lactate (Coco et al., 2013).

The first demonstration of a signaling pathway involving lactate and receptor interaction in the brain was demonstrated by Bozzo et al. (2013), who showed that L-lactate modulated the calcium spike frequency in primary mouse neuron cultures. The authors showed, for the first time, a brain non-energetic effect of L-lactate via HCAR1, since other metabolic substrates, such as pyruvate or glucose, could not mimic this effect of L-lactate. In addition, they used the agonist DHBA, reproducing the effects of lactate, and D-lactate, which has a poor affinity for the monocarboxylate transporter 2 (MCT2) and works as a partial agonist. On the other hand, a non-metabolic astrocyte-neuron signaling modulation by lactate, through a different and unknown mechanism, was demonstrated by Tang et al. (2014). This investigation elegantly showed the release of L-lactate by astrocytes employing *in vitro* optogenetics. They also demonstrated exogenous lactate in cultured and acute brain slices and showed that *in vivo* lactate administration modulates the excitability of noradrenergic neurons from the locus coeruleus. The authors suggested a possible receptor, other than HCAR1, since D-lactate acted as an inverse agonist and lactate concentrations used were about ten times lower than the IC50 for the G_i_-coupled receptor.

A study revealed L-lactate upregulation of immediate early genes associated with *N*-methyl-D-aspartate (NMDA) transmission in neuronal cultures from the mouse neocortex and *in vivo* administration of L-lactate (Yang et al., 2014). Genes such as Arc, c-Fos, and Zif268 had an increased expression after lactate treatment in a range between 2.5 and 20 mM in a time-dependent manner, with a one-hour peak. An energetic effect was excluded after the ineffectiveness of D-lactate, pyruvate, and glucose in an equicaloric concentration at the same experimental conditions. Moreover, signaling of lactate on these plasticity-related genes was intracellular, since the MCT blocker UK5099 abolished this effect. Interestingly, after longer treatment periods, lactate also stimulated an increase in BDNF expression and the phosphorylation of Erk1/2. The authors showed lactate action via NMDA receptors, but not a specific lactate receptor.

More recently, one study showed the modulation by lactate of the action potential frequency in pyramidal cells from the CA1 region of the hippocampus, under stable energetic conditions. Both lactate and its agonist, DHBA, induced a biphasic modulation in neuronal excitability, inducing reduced excitability at lower concentrations (lactate at 5 mM and DHBA at 0.56 mM), while higher concentrations (lactate at 30 mM and DHBA at 3.1 mM) increased firing frequencies. Use of a neuronal MCT2 blocker did not abolish the lactate effect and neither did D-lactate alter the firing frequency of the cells; however, G_i_ protein inhibition via pertussis toxin confirmed the effect of lactate via HCA1R (Herrera-López and Galván, 2018).

Lactate release in response to glutamate uptake was described 20 years ago (Pellerin and Magistretti, 1994); however, an alternative molecular pathway for lactate efflux, induced by neuronal depolarization has been proposed (Choi et al., 2012). A soluble adenylyl cyclase (sAC) sensitive to HCO_3_^-^ is found abundantly expressed in astrocytes and responds to extracellular K^+^ elevation. An increase in cAMP secondary to HCO_3_^-^influx (via HCO_3_^-^/Na^+^ transporter) was observed in cultured astrocytes and in brain slices; furthermore, sAC was found to be responsible for the production and release of lactate as a consequence of the glycogen breakdown coupled with K^+^ increase in astrocytes (see Figure 2). As cAMP levels stimulate glycogen breakdown (Pellerin et al., 2007), HCA1 receptor (coupled to G_i_ protein) activation via lactate could mediate glycogenolysis feedback control by lactate in astrocytes.

The lactate response during neurotransmission is fast and independent of the metabolic status or oxygen availability, leading to the observation by Sotelo-Hitschfeld et al. (2015) of a steady-state reservoir of lactate (Sotelo-Hitschfeld et al., 2015). The synaptic activity and consequent depolarization caused by extracellular K^+^ have been reported to elicit glycogen mobilization (Choi et al., 2012) and lactate generation (Sotelo-Hitschfeld et al., 2012). On the other hand, using a FRET lactate sensor, the group demonstrated that depolarization, via depletion of the astrocytic lactate reservoir in cultured astrocytes, may occur via a non-identified anion channel (Sotelo-Hitschfeld et al., 2015).

HCAR1-mediated lactate effects have been suggested to be neuroprotective in depression (Carrard et al., 2018) and cerebral ischemia (Berthet et al., 2009, 2012). More recently, in a middle cerebral artery occlusion stroke model, HCAR1 receptor expression was increased in the hippocampus, the cortex, and the striatum after ischemia (Castillo et al., 2015). Moreover, in hippocampal slices in an oxygen and glucose deprivation model, DHBA and D-lactate protected the CA1 region from the insult (Castillo et al., 2015). Although it is not possible to determine the definitive role for HCAR1 in the synaptic communication at this moment, the astrocyte lactate released during glycogenolysis is reportedly crucial for memory consolidation (see Hertz and Chen, 2018 for review). However, recent data indicate that an aging-associated shift of glycogen metabolism enzyme concentrations, and their localization in astrocytes and neurons, may occur (Drulis-Fajdasz et al., 2018). In addition to its role in synaptic signaling modulation, the astrocytic steady-state reservoir, and the rapid response to depolarization, lactate may act as a gliotransmitter molecule (Tang et al., 2014; Sotelo-Hitschfeld et al., 2015). However, synthesis rather than release should be considered as the limiting step for lactate signaling in the brain (Mosienko et al., 2015).

### Methylglyoxal and D-Lactate Also Affect Neurotransmission

**FIGURE 3:**
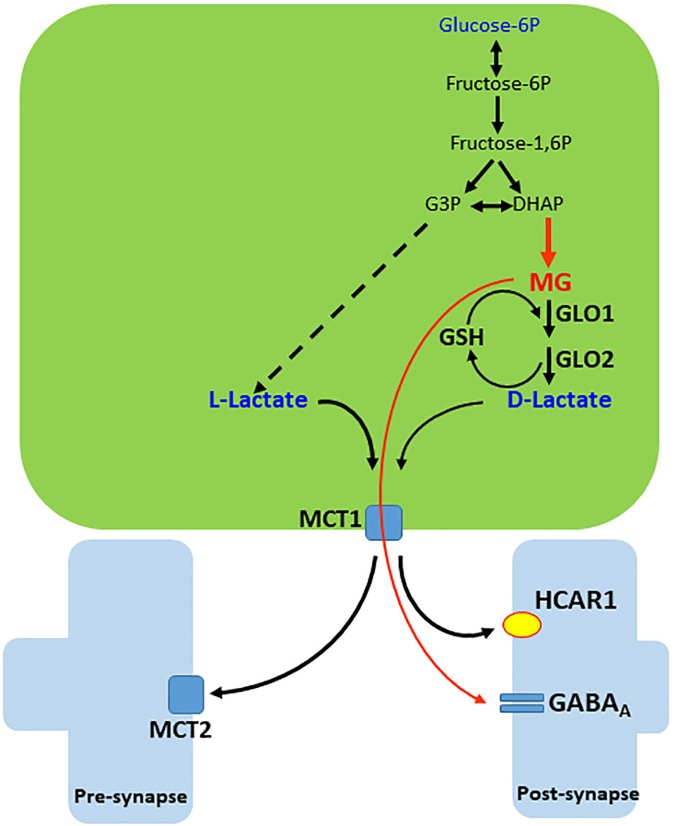
Generation and release of MG and D-lactate in astrocytes. Methylglyoxal (MG) is produced from dihydroxyacetone phosphate (DHAP) by a deviation of the glycolytic pathway. MG is condensed with glutathione (GSH) and then, by sequential action of glyoxalases 1 and 2 (GLO1 and 2), generates D-lactate and recycles GSH. D-lactate leaves the cell via monocarboxylate transporter 1 (MCT1) but disturbs lactate and pyruvate flows to mitochondria (not illustrated). MG and D-lactate leave the cell and act on the GABA_A_ receptor and hydroxy-carboxylic acid receptor 1 (HCAR1), respectively. Extrasynaptic GABA_A_ receptors of MG are not illustrated.

Methylglyoxal is a dicarbonyl compound (formula CH_3_C(O)CHO) derived from endogenous and exogenous sources and responsible for most of the glycation reactions in cell metabolism. The endogenous source of MG comes from enzymatic or non-enzymatic reactions of reducing sugars, lipids and amino acids in the cell. The main source of MG synthesis is from aldehydes, which are intermediates of the glycolysis pathway, such as glyceraldehyde 3-phosphate and dihydroxyacetone-phosphate (see Figures 1, 3). At physiological or pathological conditions, MG is produced through spontaneous dephosphorylation of dihydroxyacetone-phosphate (Angeloni et al., 2014; Muronetz et al., 2017). Coffee, alcoholic beverages, cigarette smoke and food are all exogenous sources of MG (Nemet et al., 2006; Angeloni et al., 2014).

Methylglyoxal is also present in different biological materials (tissues, urine, plasma and the cerebrospinal fluid) and its concentration is related to the status of glucose metabolism (Nemet et al., 2006; Angeloni et al., 2014). It has been suggested that about 0.1–0.4% of the glycolysis pathway results in the formation of MG (Kalapos, 2008). More recently, it has been proposed that MG, at physiological levels (μM), acts as an agonist of the γ-aminobutyric acid type A (GABA_A_) receptor and is associated with anxiolytic behavior (Distler et al., 2012) and induction of sleep (Jakubcakova et al., 2013). However, due to the lower levels of MG in the synaptic cleft (compared to those of GABA), it has been proposed that MG has a modulatory effect on GABA_A_ receptors in the extrasynaptic space (Tao et al., 2018).

On the other hand, the relationship between GABA_A_ and glucose metabolism (more precisely, glucose uptake) has been investigated over the last 30 years, but findings are not clear and sometimes conflicting (Ito et al., 1994; Peyron et al., 1994; Parthoens et al., 2015). Currently, measurement of glucose metabolism (based on deoxyglucose uptake) is associated with glutamatergic activity, mainly because glutamate is the predominant neurotransmitter. However, other neurotransmitters such as noradrenaline and adenosine, as well as K^+^ itself, released by neuronal activity, modulate energetic metabolism (Hertz et al., 2015; Waitt et al., 2017). In this scenario, MG is a glucose-derivative molecule that putatively connects energetic metabolism and the GABAergic system.

Methylglyoxal is metabolized predominantly by the cytoplasmic glyoxalase system, formed by two enzymes, glyoxalases 1 and 2 (GLO1 and 2), which act sequentially. GLO1 depends on GSH. GSH reacts directly with MG and produces hemithioacetal and GLO1 converts this metabolite to S-Lactoylglutathione. Subsequently, this compound is hydrolyzed by GLO2 into D-lactate and GSH is regenerated (Figure 3). Notice that, due to the lower activity of the left leg of the destination of glucose in neurons, it may be assumed that extracellular MG and D-lactate, as well as L-lactate, originate predominantly from astroglial cells.

D-lactate is oxidized to pyruvate by a D-isomer-specific lactate dehydrogenase (D-LDH; Cristescu et al., 2008). Mitochondrial D-LDH activity in rat brain tissue is about 60% lower than in liver (Ling et al., 2012). High levels of D-lactate inhibit the membrane L-lactate transport and pyruvate transport to mitochondria in astrocytes (Gibbs and Hertz, 2008) and neurons (Ros et al., 2001), which explains the neurotoxicity of this compound. However, a neuroprotective effect of D-lactate has been proposed in seizures (Angamo et al., 2017), possibly due to energy impairment. More recently, in addition to confirming the presence D-LDH activity in mouse brain tissue, it was shown that D-lactate is a partial agonist of HCAR1 (Castillo et al., 2015).

### Astrocyte Dysfunction, MG-Induced Protein Glycation, and Neurodegenerative Diseases

It is well known that in aging, diabetes mellitus, and neurodegenerative diseases, MG is elevated to sub-millimolar levels, working as a potent glycant agent (Srikanth et al., 2013; Maessen et al., 2015). Elevated D-lactate levels from liver metabolism are observed in diabetic animals (Kondoh et al., 1994) and the accumulation of this compound could contribute to memory impairment, dependent on lactate flow (Suzuki et al., 2011). However, when the detoxifying system fails due to a reduction in glyoxalase activity or GSH deficiency, MG and advanced glycation end-products (AGEs) formation increases, but D-lactate levels can be reduced, as has been observed in endothelial cells (Li et al., 2013).

Methylglyoxal promotes glycation on lipids, nucleic acid and proteins (Allaman et al., 2015). MG mainly promotes post-translation modifications on proteins by the Maillard reaction on amino acid residues. The most common glycated amino acids are arginine and lysine and consequently the formation of AGEs such as argpyrimidine, hydroimidazolone (MG-H1), Nε-(1-carboxyethyl)-L-lysine (CEL), and Nε-(1-carboxymethyl)-L-lysine (CML) adducts, respectively (Rabbani and Thornalley, 2012). AGEs are ligands of the receptor for advanced glycation end products (RAGE) and induce inflammation by activation of the nuclear factor κB (NF-κB) pathway in all neural cells (Chavakis et al., 2004; Donato et al., 2009).

Elevated MG seems to increase the expression of astrocyte markers (glial fibrillary acidic protein – GFAP and S100B) and cytokines in astrocyte culture and *in vivo*, leading to astrogliosis and neuroinflammation (Chu et al., 2016). However, cognitive impairment has also been reported without changes in classical parameters of astrogliosis (Hansen et al., 2016b). Impairment in glucose flow and/or dysfunction of the glyoxalase system is a common and early event in neurodegenerative diseases, such as Alzheimer’s (AD) and Parkinson’s diseases (PD).

Elevated MG and AGEs levels play a key role in protein misfolding and oxidative stress, and are also involved in AD (Angeloni et al., 2014). The increases in glycation reactions and MG levels are suggested as a possible link in diabetic individuals that develop AD (Janson et al., 2004). In different *in vitro* models, MG induced glutamatergic excitotoxicity by promoting glutamate release (Arriba et al., 2006) and glutamate uptake disturbance (Hansen et al., 2016a, 2017). Elevated MG and AGEs stimulate apoptosis and reduce neurogenesis as well as neuronal survival in the hippocampus by downregulation of BDNF expression and its signaling pathways (Di Loreto et al., 2008; Falone et al., 2012; Chun et al., 2016). MG is involved in tau hyperphosphorylation through activation of GSK-3β and p38 mitogen-activated protein kinases (p38 MAPK) (Li et al., 2012). Moreover, AGEs and hyperphosphorylated tau are co-localized in the cytoplasm of neurons, possibly contributing to neurofibrillary tangle formation (Lüth et al., 2005; Fawver et al., 2012). AGE immunoreactivity has also been observed in amyloid plaques (Münch et al., 1997; Krautwald and Münch, 2010); recently, MG was shown to glycate Lys-16 and Arg-5 residues on β-amyloid, resulting in glycated Aβ (Fica-Contreras et al., 2017). The Aβ-AGE form is more insoluble, neurotoxic and resistant to protease reactions (Angeloni et al., 2014).

In PD, a predictor event is the low activity of neuronal PPP enzymes (Dunn et al., 2014) and mitochondrial dysfunction (Dranka et al., 2012; Hipkiss, 2014), which changes the redox cell status and increases the anaerobic glycolysis pathway and MG formation. In addition, dysregulation of the glyoxalase system (Joe et al., 2018) also leads to high MG levels and an increase in glycation reactions. MG reacts directly with dopamine, reducing its concentration and generating the salsolinol-like compound, 1-acetyl-6.7- dihydroxy-1,2,3,4-tetrahydroisoquinoline, which promotes mitochondrial dysfunction (Hipkiss, 2014). Moreover, the glycation of the N-terminal region of α-synuclein reduces its ability to remain attached to the plasma membrane. In fact, the accumulation of aggregated glycated-α-synuclein in the cytoplasm results in neurotoxic effects on dopaminergic neurons (Vicente Miranda et al., 2016).

## The Right Arm Modulates Glutathione Homeostasis and Glutamatergic Neurotransmission

**FIGURE 4:**
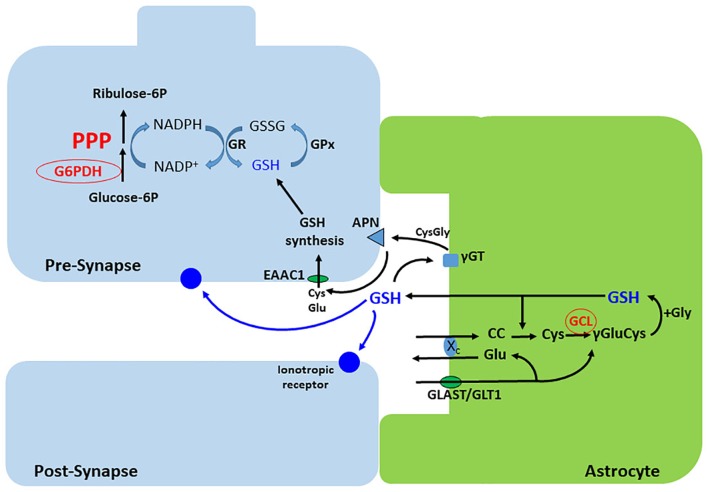
Synthesis and release of GSH in astrocytes. Cystin (cisteinyl-cystein, CC) is uptaken via the xc^-^ exchanger, which releases glutamate. This exchanger is functionally coupled to the Na^+^- dependent glutamate transporters, GLAST or GLT-1. Cystin is reduced by GSH to two cysteines. The gamma acid group of glutamate then condenses with the amine group of cysteine, forming gamma-glutamyl-cysteine (γ Glu-Cys), by the action of the glutamate cysteine ligase (GCL). Addition of glycine completes the synthesis of GSH that, in part, is exported to modulate ionotropic receptors and as a source of cysteine for GSH synthesis in neurons. The extracellular cysteine from astroglial GSH is generated after sequential action of the extracellular peptidases, γGT and APN. Neurons synthesize low levels of GSH compared with astrocytes, but exhibit a high capacity of GSH regeneration, which depends on NADPH synthesis in the PPP. γGT, gamma peptidyl transpeptidase; APN, aminopeptidase neuronal; GPx, glutathione peroxidase; GR, glutathione reductase; EAAC, excitatory amino acid carrier.

Glucose metabolism, in addition to producing energy, supports other important functions, for example the generation of reducing equivalents for antioxidant defenses and biosynthetic pathways, in both astrocytes and neurons (Magistretti and Allaman, 2018). The PPP, which constitutes the right arm of the glucose destinations (Figure 1), is the main cytosolic source of NADPH and is essential for regeneration of GSH. This pathway decarboxylates glucose-6-phosphate into ribulose-5-phosphate, a precursor for the nucleotide biosynthesis, conserving the redox energy as NADPH. Glucose-6-phosphate dehydrogenase (G6PD) is the rate-limiting enzyme for the PPP (see Figures 4, 6), which in the resting brain represents a minor pathway for glucose metabolism (Gaitonde et al., 1983; Wamelink et al., 2008; Bouzier-Sore and Bolaños, 2015). However, under several conditions, for example in response to injury, the PPP can be markedly upregulated (Bartnik et al., 2005; Jalloh et al., 2015; Rosa et al., 2015) and has demonstrated protective roles because it provides precursors for tissue repair, as well as increases GSH to avoid oxidative stress and neuroinflammation.

In astrocytes, the PPP is essential for maintaining an adequate pool of reduced GSH, since these cells export this antioxidant molecule to neurons. Activation of the PPP in astrocytes protects neurons from oxidative stress by increasing astrocytic GSH levels (Takahashi et al., 2012). In neurons, the low expression of PFKFB-3 (Figure 6) results in a low glycolytic rate, and glucose is diverted to the PPP (Almeida et al., 2004; Herrero-Mendez et al., 2009). This is particularly important for generating GSH, since neuronal cells have a lower synthesis and, consequently, GSH concentrations, compared to astrocytes (Makar et al., 1994; Dringen et al., 2005).

Glutathione is an essential molecule for cellular antioxidant defense and detoxification processes, conferring neuroprotection (Dringen, 2000b; Dringen et al., 2015; Kinoshita et al., 2018). Impaired GSH metabolism is associated with oxidative stress and inflammatory responses, which have been linked to cerebral diseases and neurodegeneration (Dringen, 2000a; Lee et al., 2010; Aoyama and Nakaki, 2013; Arús et al., 2017). An age-dependent reduction in resting NADPH concentration, accompanied by a decrease in GSH levels, has been reported in cultured neurons, making them more susceptible to glutamate exposure (Parihar et al., 2008). This impaired reducing power can lead to pathological aging, since the inability of neurons to regenerate GSH is a hallmark of AD and PD (Currais and Maher, 2013).

Glutathione is a tripeptide, consisting of glutamate, cysteine and glycine, that is synthesized by two enzymatic steps. First, GCL catalyzes the reaction between glutamate and cysteine to form the dipeptide γ-glutamylcysteine. The second step is the reaction of GSH synthase, which mediates the addition of glycine to γ-glutamylcysteine to form GSH (Lu, 2013). While the first step catalyzed by GCL is considered the rate-limiting reaction, the intracellular content of cysteine is the rate-limiting precursor for GSH biosynthesis (Griffith, 1999; Figure 4).

Glutathione in the brain is present in both intra- and extracellular compartments and acts as a dynamic buffer of the redox state. Non-enzymatically, GSH is able to react directly with free radicals including superoxide, hydroxyl radical, nitric oxide, peroxynitrite and MG (Clancy et al., 1994; Winterbourn and Metodiewa, 1994; Aoyama and Nakaki, 2015). Moreover, GSH can react with protein thiol groups, leading to a reversible formation of mixed disulfides (S-glutathionylation), which are important for preventing protein oxidation, thus preserving and modulating its functions (Giustarini et al., 2004). GSH also participates in enzymatic reactions, such as those catalyzed by GSH peroxidase (GPx) and glutathione-S-transferase (GST). GPx detoxifies hydrogen peroxides and other endogenous hydroperoxides using GSH as an electron donor. In this reaction, GSH is oxidized to glutathione disulfide (GSSG; Dringen et al., 2015); GSSG is then reduced back to GSH via glutathione reductase (GR) using NADPH (Dringen and Gutterer, 2002; Ren et al., 2017).

The high oxidative metabolic rate can increase mitochondrial reactive oxygen species (ROS) production, rendering the brain vulnerable to oxidative stress. Astrocytes and neurons exhibit differences in GSH metabolism and different mechanisms maintain GSH homeostasis between these cell types. In astroglial cells, the basal levels of GSH are higher than in neuronal cells, showing their pivotal antioxidant role in the central nervous system (Dringen et al., 2005). Neurons, in turn, depend on astrocytic GSH release for providing extracellular cysteine for their synthesis of GSH (Figure 4). As previously mentioned, cysteine is the limiting precursor for synthesis of GSH.

Astrocytes have a Cys-Glu exchanger (system xc^-^) that mediates the uptake of cystine, the bioavailable form of cysteine, in exchange for glutamate (Bridges R.J. et al., 2012; Ottestad-Hansen et al., 2018). Moreover, they express the glutamate/aspartate transporter GLAST (also known as excitatory amino acid transporter 1, EAAT1 in humans) and glutamate transporter 1 (GLT1, or EAAT2 in humans) (Lehre and Danbolt, 1998), which provide intracellular glutamate for GSH synthesis and for system xc^-^ operation (Reichelt et al., 1997). Importantly, these glutamate transporters also account for the majority of glutamate removal from the synaptic cleft, maintaining extracellular glutamate concentrations low to avoid excitotoxicity (Anderson and Swanson, 2000; Rose et al., 2017). In this regard, system xc^-^ and glutamate transporters are associated with both GSH biosynthesis and modulation of glutamatergic neurotransmission, as xc^-^ mediates glutamate release. Altered function of these transporters can result in GSH depletion and/or, consequently, glutamate excitotoxicity in pathological conditions (Ré et al., 2003; Yi and Hazell, 2006; Bridges R. et al., 2012; Thorn et al., 2015). At the same time, GLAST/GLT1 are also related to glucose metabolism; as they are sodium-dependent, their activity increases the intracellular sodium concentration, consequently activating the Na^+^/K^+^ ATPase pump, which consumes ATP in astrocytes. ATP, in turn, can be supplied by the glycolytic pathway (for a review, see Bélanger et al., 2011).

Glutamate is also involved in the mechanism by which astrocytes are able to readily release GSH in response to neural activity, to maintain neuronal GSH levels via the astrocyte-neuronal GSH shuttle. In astrocytes, glutamate triggers a cascade of signals that promote the expression of antioxidant genes through activation of the nuclear factor (erythroid-derived 2)-like 2 (Nrf2), leading to the biosynthesis of GSH (Frade et al., 2008; Jimenez-Blasco et al., 2015; McGann and Mandel, 2018). In the extracellular space, GSH can be hydrolyzed by γ-glutamyl transpeptidase forming γ-glutamyl and the dipeptide CysGly. CysGly is, in turn, cleaved by the neuronal aminopeptidase N into cysteine and glycine, which serve as precursors for neuronal GSH synthesis (Dringen et al., 1999, 2001; Hertz and Zielke, 2004; Figure 4). Neuronal cells are less capable of importing cystine, but the sodium-dependent excitatory amino acid carrier 1 (EAAC1, also known as EAAT3 in humans) is able to uptake cysteine in addition to glutamate (Zerangue and Kavanaugh, 1996; Shanker et al., 2001). EAAC1/EAAT3 supplies neurons with the rate-limiting precursor for GSH synthesis, directly influencing their redox homeostasis (Paul et al., 2018). Furthermore, EAAC1/EAAT3 acts as a bridge between astrocytic and neuronal GSH metabolism by importing cysteine released from the extracellular breakdown of astrocytic GSH.

Glucose metabolism, GSH synthesis and glutamatergic homeostasis are closely associated processes and share extracellular and intracellular regulatory mechanisms. Among these, the neurotrophic factor BDNF has recently been demonstrated as a key regulator of central energy homeostasis (Marosi and Mattson, 2014). BDNF increases glucose transport in neurons by inducing the expression of GLUT3 through phosphatidylinositol-3 kinase (PI3K) and Akt kinase (Burkhalter et al., 2003). Because of the low neuronal content of PFKFB-3, glucose can be used for the generation of GSH through the PPP. Additionally, BDNF can activate hypoxia-inducible factor-1 (HIF-1) and Nrf2, which are related to the induction of enzymes that participate in glucose metabolism in both astrocytes and neurons. Importantly, Nrf2 regulates G6PD, GCL, GSH synthase, system xc^-^, and EAAC1/EAAT3 (Thimmulappa et al., 2002; Shih et al., 2003; Escartin et al., 2011; Niture et al., 2014), thus facilitating both regeneration and synthesis of GSH.

Experimental data have suggested a role for GSH as a neuromodulator. As a thiol-containing compound, GSH may regulate the redox sites of several ionotropic receptors and ion channels, altering their functional characteristics (Gozlan and Ben-Ari, 1995; Pan et al., 1995). In this regard, both GSH and GSSG have been demonstrated to modulate neuronal depolarization, calcium ion influx and second messenger activity (Janáky et al., 1993; Varga et al., 1997) through the glutamatergic receptors NMDA and α-amino-3-hydroxy-5-methylisoxazole-4-propionic acid (AMPA). Such effects appear to be dependent on GSH concentrations. At micromolar levels, GSH is inhibitory via its interaction with glutamate binding sites. In contrast, at millimolar concentrations, GSH activates NMDA receptors by reducing functional thiol groups (Janáky et al., 1993). Interestingly, as a consequence, GSH becomes oxidized to GSSG, which triggers an increase in the PPP to generate GSH (Delgado-Esteban et al., 2000).

In retinal glial cells, GSH induces calcium shifts in a P2X7 (a purinergic receptor subtype), but not ionotropic glutamate receptor dependent manner. In contrast, GSSG did not reproduce this effect, indicating that the antioxidant and/or structural features of GSH are essential to promote elevations in cytoplasmic calcium levels (Freitas et al., 2016). In cortical brain slices, GSH was able to evoke a depolarizing potential that appears to be mediated by sodium ions. As this potential was not blocked by antagonists of glutamate receptors, GSH may act through its own receptor-mediated channels (Shaw et al., 1996). In this regard, radioligand binding assays have shown the presence of binding sites for GSH in different neural cell preparations, including brain synaptosomal membranes (Janáky et al., 2000) and astrocytes (Guo and Shaw, 1992). GSH also seems to be released upon calcium-dependent depolarization in brain slices (Zängerle et al., 1992) and is able to modulate the release of neurotransmitters, including GABA (Janáky et al., 1994; Freitas et al., 2016) and dopamine (Janáky et al., 2007). Interestingly, GSH can reverse aging-associated hippocampal synaptic plasticity deficits (Yang et al., 2010). Together, these data support an emerging role of GSH in signal transduction and synaptic transmission.

## The Right Leg Controls the Other Destinations of Glucose in Neural Cells by Protein Glcnacylation

### The Protein GlcNAcylation and Hexosamine Pathway

**FIGURE 5:**
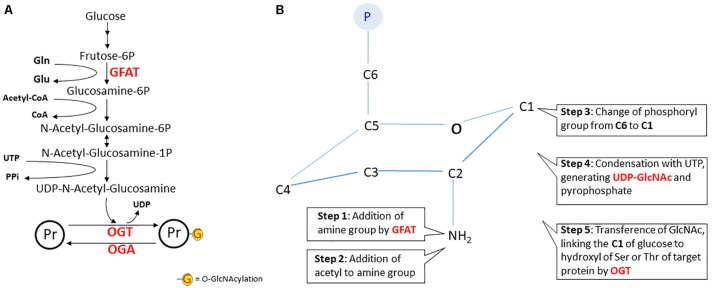
The hexosamine pathway. In panel **A**, the steps of the hexosamine pathway (HP) from frutose-6P to UDP-*N*-acetyl-glucosamine. Notice that glutamine (Gln), Acetyl-CoA and UTP are key substrates in this pathway. The rate-limiting step is catalyzed by glutamine:fructose-6P aminotransferase (GFAT). In panel **B**, a schematic representation of the structural changes during *N*-acetyl-glucosamine synthesis. The first reaction is the amination of fructose-6P (fructofuranose) to glucosamine-6P (glucopyranose). It is worth noting that, in step six, carbon 1 of glucopyranose binds to the hydroxyl of serine or threonine of the protein target. In fact, this is a reaction of *O*-linked β-*N*-acetylglucosaminylation, but for simplification, the nomenclature widely used is O-GlcNAcylation. However, this gives the wrong idea that linking occurs at the acyl group. It would be better to use NAGylation, since NAG is another (and the simplest) abbreviation of *N*-AcetylGlucosamine, found in some polymers. Herein, we will maintain the use of the term “O-GlcNAcylation.” Abbreviations: OGT, O-GlcNAc transferase; OGA, O-GlcNAcase; Pr, protein.

The post-translational modification of proteins by *O*-linked-*N*-acetyl-D-glucosamine (O-GlcNAc) is assumed to be a glucose-responsive mechanism that modulates cellular signaling (see Nagel and Ball, 2014 for a review). O-GlcNAc rapidly cycles onto the serine or threonine residues of target proteins. This process is equivalent to phosphorylation and occurs via the activity of two enzymes: O-GlcNAc transferase (OGT) and O-GlcNAcase (OGA), which add and remove GlcNAcetyl, respectively (see Figure 5). GlcNAcetyl is derived from the hexosamine biosynthetic pathway (HP), the right leg in our X of metabolic glucose destination. It is estimated that 2–5% of incoming glucose goes to the HP, which has UDP-GlcNAc as its final product, the donor of GlcNAc (Ozcan et al., 2010). In the liver, the levels of O-GlcNAc-modified proteins fluctuate according to the nutrient status, i.e., they are regulated by intracellular concentrations of UDP-GlcNAc that rise with feeding and are increased in diabetes mellitus patients (Hanover et al., 2010; Nagel and Ball, 2014). In the brain, under conditions of hypometabolism of glucose, as observed in AD, the levels of O-GlcNAc-modified proteins are reduced (Dos Santos et al., 2018). However, it is necessary to identify specific changes in protein GlcNAcylation to understand particular protein alterations in physiological and pathological conditions. Herein, we will discuss some aspects of HP regulation and specific targets of O-GlcNAcylation in astrocytes, which modulate glucose metabolism and synaptic communication.

The first reaction of HP is catalyzed by the glutamine:fructose-6P aminotransferase (GFAT) enzyme. The glutamine transfers the amine group to carbon 2 of fructose-6P, converting it to glucosamine-6P (Yuzwa and Vocadlo, 2014). Note that neurons depend on astroglial glutamine, since glutamine synthetase is a glial enzyme. In the next step, acetyl-CoA transfers acetyl to the amine group and then the phosphate from carbon 6 is transferred to carbon 1. This compound, *N*-acetyl-glucosamine-1P, reacts with UTP to release the end products UDP-GlcNAc and PPi. The rate-limiting step of HP is the reaction catalyzed by GFAT, which is negatively modulated by AMPK (Eguchi et al., 2009), like other glycolytic key enzymes such as PFK-1 and PFKFB-3. Therefore, besides glucose flow, GlcNAcylation of proteins is regulated by GFAT, and also by the activities of OGT and OGA (Worth et al., 2017).

The OGT and OGA enzymes are evolutionarily well conserved and have many targets involved in signal transduction, transcription, translation, cell cycle control and apoptosis. However, since these are just two enzymes (in contrast to the hundreds of protein kinases and phosphatases), there is still little understanding of how their targets are recognized (Yang and Qian, 2017). Moreover, Ser and Thr sites for GlcNAcylation and phosphorylation co-exist in the same protein and these modifications often share the same sites, establishing a complex functional relationship (Wang et al., 2007; Hu et al., 2010).

### Glucose Flow Regulates Its Own Fate and Its Derivatives

**FIGURE 6:**
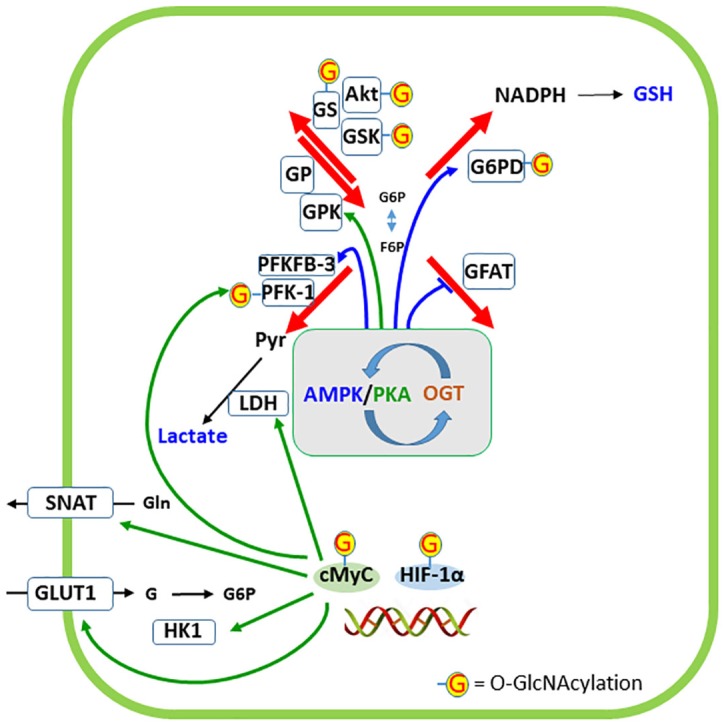
Regulation of cellular destinations of glucose by O-GlcNAc modification of proteins. AMP-activated protein kinase (AMPK) and protein kinase A (PKA) have a narrow interaction in the regulation of glucose metabolism as well as phosphorylate and are targets of O-GlcNAc transferase (OGT). The phosphorylation of specific targets of AMPK or PKA in the four cellular destinations of glucose are indicated by the respective colored arrows. Targets of OGT are indicated by G. Two transcription factors closely related to glucose metabolism (cMyc and HIF-1α) are represented at the double-strand DNA and five proteins whose expressions are regulated by these transcription factors are indicated. G6PD, glucose-6-phosphate dehydrogenase; GFAT, glutamine:fructose-6P aminotransferase; GLUT1, glucose transporter 1; GP, glycogen phosphorylase; GPK, glycogen phosphorylase kinase; GS, glycogen synthase; GSK, glycogen synthase kinase; HIF-1α, hypoxia-inducible factor-1α; HK1, hexokinase 1; LDH, lactate dehydrogenase; PFK-1, phosphofructokinase 1; PFKFB-3, 6-phosphofructo-2-kinase/fructose-2,6-bisphosphatase-3; SNAT, sodium-neutral amino acid transporter (type 3 or 5, in astrocytes).

The main protein targets of O-GlcNAcylation, which modulate glucose flow and/or destination, are indicated in Figure 6. GFAT, the regulatory enzyme of HP, is inhibited by phosphorylation, catalyzed by AMPK, at Ser 243 (Eguchi et al., 2009). Therefore, activated AMPK decreases UDP-GlcNAc levels. However, AMPK is also able to phosphorylate (activate in this case) the OGT at Thr 444, which in turn could lead to O-GlcNAcylation of AMPK, resulting in a complex regulation that involves changes in the activities of enzymes as well as their cellular localizations (Bullen et al., 2014). It is possible, but has not yet been determined, that this triangular interaction among AMPK, GFAT, and OGT occurs in neural cells, particularly in astrocytes, where AMPK has a crucial role (Bolaños, 2016).

Glutathione, as discussed above, has an important antioxidant role in neurons. However, at higher concentrations in astrocytes, it is exported not only to support neuronal synthesis, but also to modulate ionotropic synaptic receptors. Glucose generates NADPH in the PPP to recycle GSH in neurons and astrocytes. The G6PD enzyme is the rate-limiting step of this pathway. G6PD is modulated by O-GlcNAcylation, as demonstrated in several cell lines (Rao et al., 2015). In contrast to O-GlcNAcylation of GFAT, this modification of G6PD activates the enzyme, increasing the activity of the PPP and NADPH formation. Although this is of importance in neurons to regenerate GSH, the effect of O-GlcNAcylation on neuronal G6PD has not yet been analyzed. However, it is possible to realize the importance of glutamine from astrocytes to neuronal synthesis of glucosamine-6P, the precursor of UDP-GlcNAc. Another important aspect of GSH synthesis, particularly in astrocytes, is its modulation by AMPK (Guo et al., 2018). AMPK positively regulates the expression of the modulatory subunit of enzyme GCL through the transcriptional co-activator peroxisome proliferator-activated receptor gamma coactivator 1-alpha (PGC-1α), (Figure 4), which catalyzes the first step (and regulatory step) of GSH synthesis.

Glycogen synthase (GS) is covalently modified by phosphorylation (induced by catecholamines and insulin) and O-GlcNAcylation, at least in adipocytes (Parker et al., 2004). Phosphorylated GS is less sensitive to the allosteric activator, glucose-6P. Insulin phosphorylates both GSK3 and the glycogen-targeting protein through the PI3K/Akt pathway, in turn activating protein phosphatase 1, PP1. Phosphorylation of both GSK-3 and PP1 activates GS, which leads to glycogen formation (Obel et al., 2012). Similarly, glucose flow in the HP leads to O-GlcNAcylation and activation of the GS. This is an example of mutual exclusivity, where O-GlcNAcylation acts similarly to dephosphorylation, which cannot be generalized to other conditions. Moreover, this relationship is more complex because the upstream enzymes, Akt and GSK3, are also targets of O-GlcNAcylation (e.g., Park et al., 2005).

Glycogen breakdown is also regulated by phosphorylation of glycogen-targeting protein by protein kinase A (PKA) at a different site of Akt (Bak et al., 2018). This PKA-induced phosphorylation is triggered by neurotransmitters. The resulting phosphorylation of glycogen-targeting protein at the glycogen granule dissociates PP1, glycogen phosphorylase (GP), glycogen phosphorylase kinase (GPK), and GS. PKA phosphorylates/activates GPK, which in turn phosphorylates/activates GP. This dissociation is an important step because both GP and GPK are targets of PP1. Moreover, PKA phosphorylates/activates the inhibitor 1 of PP1. It was recently reported that the catalytic subunit of PKA is a target of O-GlcNAcylation (Xie et al., 2016), but the direct effect on glycogen breakdown remains unclear. Interestingly, O-GlcNAc protein modification increases in tumor cells, in response to glucose deprivation, through glycogen degradation (Kang et al., 2009), contradicting the idea that an increase in O-GlcNAcylation acts as a negative feed-back signal to ATP generation from glucose. This phenomenon may involve changes in GS/GP balance, due to changes in OGT and/or OGA, and not UDP-GlcNAc levels (Taylor et al., 2008).

Lactate generation is strongly regulated by O-GlcNAcylation because PFK-1 and PFKFB-3 are direct and indirect targets of OGT, respectively. O-GlcNAc modification of PFK-1 at Ser529 is induced by hypoxia in cancer cells and this modification inhibits enzyme activity and redirects the flux of glucose to PPP (Yi et al., 2012). The authors also observed a modest O-GlcNAcylation of HK. PFKFB-3, which regulates PFK-1, is phosphorylated by AMPK and Akt, which are targets of OGT, as mentioned above. Therefore, it is possible to conceive an interaction between AMPK, PFKFB-3, and OGT, that is just as complex or even more than the interaction between AMPK-GFAT-OGT (Bullen et al., 2014). Moreover, at least two transcription factors that have been well studied in tumor cells and are connected to glycolysis are modified by O-GlcNAcylation: c-Myc and HIF-1α. Akt/c-Myc activation induces expression of GLUT-1, HK 1 and 2, PFK-1, lactate dehydrogenase A and glutamine transporters (Miller et al., 2012; Swamy et al., 2016; Zhang et al., 2017). HIF-1α induces GLUT-1 and 3, hexokinases, and PFK-1 (Chen and Russo, 2012). Interestingly, also in cancer cells, lactate is able to trigger changes in glutamine uptake and metabolism (Pérez-Escuredo et al., 2016), which are necessary not only for cell proliferation but also for protein O-GlcNAcylation. Considering the importance of glutamine/glutamate in brain tissue and lactate in neuron/astrocyte communication, it would seem that this mechanism is worthy of investigation in the nervous system.

Finally, the interplay between AMPK and PKA in glucose metabolism should be considered. Microdomains of cAMP have been characterized to explain the localized action of this intracellular messenger, at the membrane or on soluble adenylyl cyclase. cAMP acts on PKA and is inactivated by phosphodiesterase. Both enzymes (PKA and sAC) bind to A-kinase anchoring protein (AKAP; Zippin et al., 2004; Oliveira et al., 2010). A physical connection between PKA and AMPK, via AKAP, has been proposed in muscle cells, where AMPK phosphorylates AKAP, releasing PKA (Hoffman et al., 2015). In hepatocytes, PKA phosphorylates and reduces AMPK activity (Hurley et al., 2006). Again, although most data regarding protein O-GlcNAcylation derive from tumor and/or non-neural cells, protein O-GlcNAcylation may also occur in the brain. Therefore, it is possible that a general cross-talk occurs between PKA and AMPK (involving O-GlcNAcylation, because both are targets of OGT) with a role in the metabolic regulation of glucose destination and synaptic plasticity.

## Summary

The importance of glucose for brain activity is very clear, since glucose provides ATP and replenishment of substrates, such as glutamate and cholesterol. Additionally, glucose metabolism provides derivatives such as lactate, MG and GSH, which regulate synaptic communication. Herein, we propose an intersection in an “X” that defines the four destinations of glucose in neural cells, where astrocytes work as integrative and modulatory elements in the synaptic communication. Such destinations depend on the metabolic arrangement in each cell type, which in turn depends on the glucose supply and neural activity. Extracellular L-lactate released by astrocytes, either generated from recently captured glucose or from glycogen, binds to HCAR1, a specific perivascular and post-synaptic receptor, regulating synaptic plasticity. Currently, lactate is being considered as a putative gliotransmitter. MG results from a deviation of the glycolytic pathway and is metabolized to D-lactate. Both MG and D-lactate are released and modulate neuronal activity, possibly through GABA_A_ and HCAR1, respectively. The main cellular antioxidant GSH, whose regeneration depends on the PPP, is also released by astrocytes and alters the synaptic response by modulating the redox and non-redox sites of ionotropic receptors and ion channels. O-GlcNAcylation is an important post-translational protein modification for cell signaling, and the glucose flow through the HP regulates the content of UDP-GlcNAc. The levels of UDP-GlcNAc, in part, are determinant for the GlcNAc-modification of proteins, including the proteins that modulate the glucose destinations. As discussed above, glutamine from astrocytes is essential, literally, for the neuronal synthesis of UDP-GlcNAc. Although the coupling between astrocytes and neurons most often addresses the relationship of glutamate or GABA with the glycolytic pathway, we should not forget that other neurotransmitters also modulate glucose metabolism, which then regulates neurotransmission through glycolysis-derived products.

## Author Contributions

All authors listed have made a substantial, direct and intellectual contribution to the work, and approved it for publication.

## Conflict of Interest Statement

The authors declare that the research was conducted in the absence of any commercial or financial relationships that could be construed as a potential conflict of interest.
